# Development of Cross-Scale Structured Hybrid Fiber-Reinforced Shotcrete

**DOI:** 10.3390/ma19143102

**Published:** 2026-07-19

**Authors:** Mengmeng Liu, Lu Zhang, Xiaoou Zhang, Wenwen Xing, Wenhua Zhu, Huadong Li, Zhiqiang Chen, Zhongjing Hu

**Affiliations:** 1 School of Architecture and Civil Engineering, Xihua University, Chengdu 610039, China; 0720090008@xhu.edu.cn (M.L.); 212024085900035@stu.xhu.edu.cn (X.Z.); 212024085900068@stu.xhu.edu.cn (W.X.);; 2 College of Intelligent Construction and Environmental Engineering, Chengdu Qinggong Polytechnic University, Chengdu 611731, China; 3 Sinohybro Bureau 14 Co., Ltd., Kunming 650041, China; 4 School of Architecture and Civil Engineering, Chengdu University, Chengdu 610106, China; 5 School of Civil Engineering, Southwest Jiaotong University, Chengdu 610031, China

**Keywords:** cross-scale structured hybrid fiber-reinforced shotcrete, alkali-resistant glass fiber, carbon nanotubes (CNTs), orthogonal experiment, mechanical properties, microstructure

## Abstract

With the increasing demand for tunnel construction under extreme geological conditions such as high geo-stress, rock bursts, and fault zones, the performance requirements for shotcrete in initial support systems have become more stringent. This study develops a cross-scale structured hybrid fiber-reinforced shotcrete by incorporating alkali-resistant glass fibers including HP and HD types with different lengths and carbon nanotubes (CNTs) into a conventional shotcrete matrix. An orthogonal experimental design at four factors and four levels was adopted to investigate the effects of fiber and CNT contents on the mechanical properties and microstructure of shotcrete. Uniaxial compressive strength, splitting tensile strength, slumping, rebound rate, and microscopic characteristics such as SEM were evaluated at 3, 7, and 28 days. Results show that the optimal mix proportion is 4% HP fiber (24 mm), 2% HD fiber (18 mm), 2% HD fiber (6 mm), and 0.2% CNT. Under this formulation, the 28-day compressive and splitting tensile strengths reached 43.53 MPa and 4.85 MPa, respectively, with a rebound rate as low as 3.85%. The enhanced performance is attributed to the multi-scale reinforcement mechanism. Long fibers suppress macroscopic cracks, short fibers bridge micro-cracks, and CNTs densify the interfacial transition zone. This study provides a parametric reference for the development of high-performance shotcrete and its engineering application in complex underground excavations.

## 1. Introduction

As underground engineering increasingly extends into high-altitude, deeply buried, and geologically unfavorable regions, a growing number of tunnel projects are encountering severe disasters during construction, such as rockbursts, high geo-stress, seismic fault zones, and large deformations [1,2,3]. These challenging conditions pose significant threats to the stability and safety of tunnel structures, and consequently impose higher performance demands on shotcrete used for initial support in underground engineering [4,5].

Currently, the incorporation of reinforcing materials into shotcrete to enhance its strength and durability has become a research hotspot, with most studies focusing on the addition of fibers to improve the tensile performance of shotcrete [6,7]. Wisal Ahmed investigated the effects of single and hybrid incorporation of basalt fiber, polypropylene fiber, and glass fiber on the properties of concrete [8,9,10]. Galan explored the relationship between macroscopic performance and microscopic pore structure of steel-basalt hybrid fiber-reinforced cementitious composites under high-temperature conditions, finding that hybrid fibers effectively inhibit crack propagation [11]. Hu peng proposed that graded glass fibers with a mixture of long and short fibers can synergistically suppress micro-cracks and macro-cracks, thereby enhancing tensile strength and energy absorption capacity [12]. Tong yueping demonstrated that basalt fiber at a dosage of 0.3% improves the flexural strength of shotcrete by 51.5% in a high-geothermal environment of 60 °C [13]. Wang Yiyang verified through statistical models that natural fibers can restrain plastic cracking by forming a spatial network structure, and developed a rheological model to describe their viscosity-yield stress relationship [14,15]. Jun-Mo Yang compared amorphous fibers and steel fibers, pointing out that although the former exhibit a higher rebound rate, they are suitable for corrosion-resistant rapid repair applications [16]. Regarding shotcrete admixtures, Mohammad Hossain compared the long-term flexural performance of three accelerators including aluminate, cement mineral, and alkali-free, emphasizing the advantages of alkali-free accelerators in terms of worker safety and tunnel durability [17]. Through a 20-year tunnel lining survey, found that alkali-free accelerator joints are prone to ettringite enrichment, with leaching being the primary cause of degradation in the outermost layer.

In the field of nanomaterial-reinforced concrete, Pengcheng Li focused on the distribution of multi-walled carbon nanotubes in recycled coarse aggregate shotcrete, finding that a dosage of 0.09 wt% significantly improves flexural strength and the compactness of the interfacial transition zone [10,18]. Hu et al. reported that coating different dosages of CNTs within 0–0.5 wt% on modified epoxy resin markedly enhances the corrosion resistance of basalt fiber-reinforced polymers in alkaline environments [19]. Wang et al. systematically compared the differential effects of nano-SiO_2_ and CNTs on pore structure modification in ultra-high performance cementitious composites, demonstrating that nano-SiO_2_ reduces the most probable pore size by 40.9%, whereas fine and short CNTs more significantly reduce the total porosity by 31.9% [20]. Deng et al. found that 0.1 wt% industrial-grade MWCNTs increase the compressive strength of steel fiber-reinforced UHPC by 11.3%, and through cost–benefit analysis confirmed the engineering feasibility of this approach [6,21,22,23]. Wang et al. introduced CNTs into alkali-resistant glass fiber shotcrete and reported that at a dosage of 0.2 wt%, the compressive and splitting tensile strengths reached their peak values [24]. Acoustic emission monitoring indicated that 80% of the damage was in a stable shear mode [25,26,27,28]. Abedi et al. developed a green shotcrete incorporating sisal fibers, CNTs, and graphene nanoplatelets with self-sensing and self-heating functions, and through numerical simulation verified its suitability for tunnel linings under dynamic and static loads [29]. Çağlar employed a cohesive zone model to simulate the inclined pull-out behavior of steel fibers in CNT-reinforced UHPC, showing that 0.4 wt% CNTs enhance the fiber-matrix bond strength, and a parametric finite element study revealed the synergistic effect of inclination angle and CNT content [3].

In a view of above studies, it can be found that existing research on shotcrete reinforcement has mainly focused on the single or hybrid addition of fibers to enhance tensile performance, or the incorporation of a single type of nanomaterial to improve durability [28,30,31]. However, few studies have addressed the structured hybrid reinforcement of shotcrete combining fibers and nanomaterials to achieve comprehensive performance enhancement [32,33,34]. Therefore, this study adopts an orthogonal experimental method to design an orthogonal array, employing mechanical tests such as uniaxial compression and splitting tensile and microscopic characterization to determine the optimal dosages of hybrid alkali-resistant glass fibers and carbon nanotubes in shotcrete [35,36]. The findings provide parametric support and engineering references for the development of cross-scale structured reinforced shotcrete materials and for initial support in complex underground engineering projects [14,37,38].

Therefore, a four-factor, four-level orthogonal experimental design was adopted to efficiently evaluate the relative effects of the four reinforcement components and determine suitable dosage levels with a limited number of mixtures. Uniaxial compressive and splitting tensile tests were conducted to evaluate load-bearing capacity and crack resistance; slump and rebound tests were used to assess construction workability and material loss during spraying; and SEM observations were performed to identify the corresponding microstructural mechanisms [39,40]. The practical objective of this study is to develop a shotcrete mixture that can reduce cracking, seepage-related deterioration, spalling, and rebound loss, thereby improving the reliability, construction efficiency, and material utilization of initial support systems in tunnels subjected to high geo-stress and other adverse geological conditions.

## 2. Engineering Background

A plateau railway under construction in China has a tunnel section with a buried depth exceeding 1000 m and a length of 610 km. The railway alignment is characterized by intense tectonic movements and extremely complex geological conditions. During tunnel construction, prominent adverse geological problems are expected to be encountered, including high geo-stress rockbursts, large deformation of soft rocks, high ground temperature, active faults, and ultra-high-pressure water-rich fault zones. The surrounding rocks along the alignment are mainly clastic rocks, slate, phyllite, granitic magmatic rocks, and volcanic rocks, which are highly fractured with well-developed fissures.

**Figure 1:**
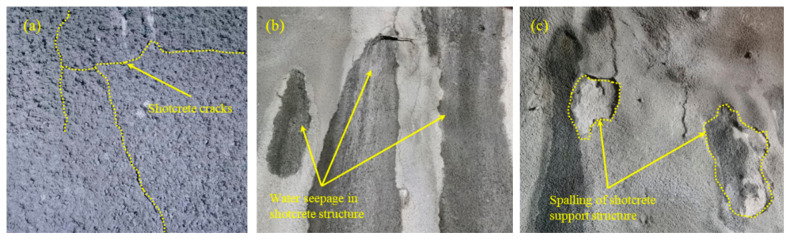
Typical failure characteristics of the shotcrete support structure in a plateau railway tunnel: (**a**) irregular cracking of shotcrete; (**b**) large-area water seepage of shotcrete; (**c**) block spalling of shotcrete.

Through field investigations and literature reviews, it was found that the shotcrete support structures in sections with high ground temperature and high geo-stress suffer from cracking, water seepage, and spalling. These damage types are mainly observed at the two sidewalls and the side walls of the tunnel, as shown in Figure 1. The three damage modes such as cracking, water seepage, and spalling are mutually correlated. Through cracks in the shotcrete can easily form channels for water seepage. When the surrounding rock is highly water-rich, groundwater seeps along these through cracks into the tunnel. At locations with high water pressure and large water inflow, extensive water immersion can be clearly observed on the shotcrete surface. After water immersion, the strength of the shotcrete itself and the bond strength between the shotcrete and the surrounding rock are significantly reduced, leading to shotcrete spalling. At the spalling positions, exposed steel reinforcement meshes can be observed. In the high-temperature and high-humidity environment, these steel meshes are highly susceptible to corrosion, thereby endangering the safety of tunnel support.

These field observations indicate that shotcrete for such tunnel sections should not be optimized solely in terms of peak compressive strength. The material must simultaneously provide sufficient tensile crack resistance, stable fresh-state workability for spraying, adequate adhesion to the surrounding rock surface, and a low rebound rate. A cross-scale reinforcement system is therefore expected to provide practical benefits by restraining crack initiation and propagation, reducing the formation of seepage channels and subsequent spalling, and decreasing material loss during spraying. These engineering requirements form the principal performance criteria for the mixture design and experimental evaluation presented in the following sections.

## 3. Experimental Program

### 3.1. Raw Materials and Mix Design

**Figure 2:**
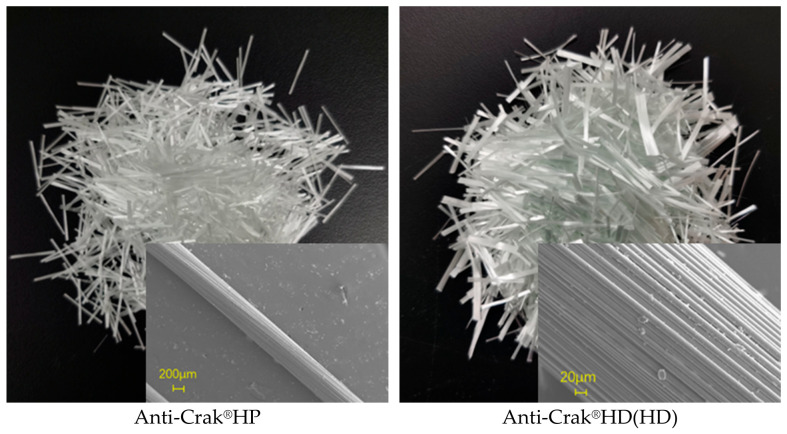
Macro and micro morphologies of alkali-resistant glass fibers.

**Figure 3:**
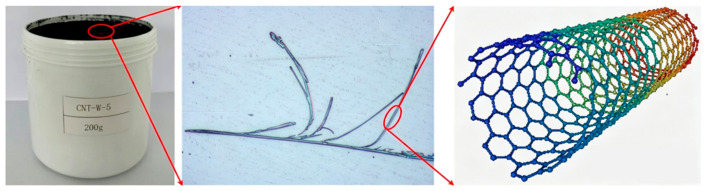
Aqueous dispersion of carbon nanotubes used in the experiment (CNT-W-5). (Caption: Name: carbon nanotube aqueous dispersion; Specification: CNT-W-5; Net weight: 200 g).

The materials used in this experiment include cement, coarse aggregate, fine aggregate, alkali-resistant glass fibers, CNTs, and water. The admixtures are superplasticizer and accelerator. The cement is P.O. 42.5 ordinary Portland cement produced by Zhonglian Cement Co., Ltd. (Beijing, China). The physical and mechanical properties of this cement are presented in Table 1. The coarse aggregate is graded crushed stone with a particle size range of 5–10 mm, a clay content ≤ 1.0%, and a mud lump content ≤ 0.5%. The fine aggregate is natural river sand with a fineness modulus of 2.9, belonging to grading zone II. The alkali-resistant glass fibers, namely Anti-Crak^®^ HD (HD) and Anti-Crak^®^ HP (HP), were supplied by Sinoma Taishan Fiberglass Co., Ltd. (Taian, China). These fibers exhibit high dispersibility, good corrosion resistance, extremely low electrical conductivity, and high chemical resistance. The fiber lengths were selected according to the requirements of graded crack control and their compatibility with the shotcrete mixture containing 5–10 mm coarse aggregate. The 24 mm HP fiber was selected as the long-fiber component because its length is substantially greater than the maximum aggregate size, enabling it to transfer stress over a relatively long distance and bridge larger cracks across the mortar–aggregate structure. The 18 mm HD fiber was selected as an intermediate-length reinforcement to control crack development between the long-fiber and short-fiber scales while maintaining better dispersibility than the 24 mm fiber. The 6 mm HD fiber, which is shorter than the maximum aggregate size, was introduced to distribute more readily within the cement paste and interfacial regions and to restrain fine and distributed cracking. The selection of the two alkali-resistant glass-fiber products was also related to the specific requirements of shotcrete construction. Unlike conventional cast concrete, shotcrete must maintain sufficient pumpability and sprayability, rapidly adhere to the substrate, limit rebound loss, and provide crack control after hardening. The selected fibers have relatively high tensile strength and elastic modulus, low density, and resistance to corrosion and alkaline environments. These properties make them potentially suitable for tunnel shotcrete exposed to humid underground conditions. The combination of the HP and HD fiber products was intended to balance long-range crack bridging with fiber dispersibility and fresh-state spraying performance. The aspect ratios of the 24 mm HP fiber, 18 mm HD fiber, and 6 mm HD fiber were calculated as 1263, 1286, and 429, respectively. The technical parameters of the fibers are listed in Table 2, and their macro/micro morphologies are shown in Figure 2. The CNTs used are an aqueous dispersion (CNT-W-5) with a concentration of 8%, produced by Beijing TanYang Technology Co., Ltd. (Beijing, China), as shown in Figure 3. The middle image in Figure 3 was obtained with a scale of 1 μm, as determined from the original image calibration. The CNTs had an outer diameter of 10–50 nm and a length of 5–20 μm, according to the manufacturer’s technical specifications. The superplasticizer and alkali-free accelerator are FZ-H100 with water-reducing rate > 35% and FZ-100C with initial setting time 160 s and final setting time 405 s, respectively, both manufactured by Shandong Fangzhou New Materials Co., Ltd. (Weifang, China). The alkali-free accelerator was used at the same dosage in all mixtures. Its principal role was to promote rapid setting and early structural build-up during shotcrete preparation. Alkali-free accelerators based mainly on aluminum salts commonly accelerate the formation of ettringite and modify the early hydration of silicate phases, thereby affecting the precipitation and spatial distribution of hydration products around aggregates and reinforcing fillers. Accordingly, the accelerator may influence the early development of the interfacial transition zones in addition to the effects produced by the fibers and CNTs. The water used in the experiments meets the requirements of tap water specified in JGJ 63-2006. The mix proportion of shotcrete is given in Table 3. The w/c ratio is calculated as 0.485.

### 3.2. Orthogonal Design

Four reinforcement-related factors, each at four dosage levels, were considered in this study. A full-factorial experimental design would therefore require 4^4^ = 256 different mixtures. Considering the large number of specimens required for mechanical testing at three curing ages, together with the workload associated with slump and field rebound tests, a full-factorial design was not practically feasible. An L16(4^4^) orthogonal array was consequently adopted to reduce the number of mixtures from 256 to 16 while maintaining a balanced distribution of factor levels. As such, in this study, an orthogonal experimental method was adopted to investigate the effects of HP fiber (24 mm length), HD fiber (18 mm length), HD fiber (6 mm length), and CNT content on the mechanical strength and microstructural characteristics of shotcrete. A four-factor, four-level orthogonal experimental scheme was designed. The HP fiber (24 mm), HD fiber (18 mm), HD fiber (6 mm), and CNT content were designated as factors α, β, γ, and δ, respectively. The four levels of factor α were 2%, 3%, 4%, and 5%. The four levels of factors β and γ were 1%, 1.5%, 2%, and 2.5%. The four levels of factor δ were 0.1%, 0.15%, 0.2%, and 0.25%. The orthogonal array of the experimental design is presented in Table 4. The dosages of HP-24, HD-18, HD-6, and CNTs are expressed as percentages by mass of the total binder. The L16(4^4^) orthogonal array was primarily selected to estimate and rank the main effects of the four reinforcement components. The 16-run design provides 15 treatment degrees of freedom, of which 12 are required for the four main effects because each four-level factor requires three degrees of freedom. A pairwise interaction between two four-level factors requires nine degrees of freedom. Therefore, the present design does not provide sufficient independent degrees of freedom to estimate the individual pairwise interactions among the three fiber components and CNTs. The interaction-related results should consequently be interpreted as possible combined non-additive effects rather than as independently quantified pairwise interactions. It should be noted that all factor levels in the orthogonal array were greater than zero. Therefore, the present design was intended to compare the relative influence of dosage variations among the four reinforcement components within the hybrid system, rather than to isolate their absolute contributions relative to unreinforced shotcrete. In particular, the independent effects of the multi-length fiber system and CNTs, as well as their interaction, cannot be fully separated without additional reference, fiber-only, and CNT-only mixtures.

### 3.3. Experimental Methods

**Figure 4:**
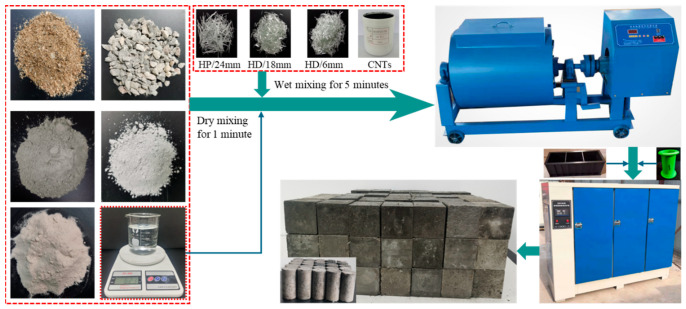
Preparation process of cubic and cylindrical specimens.

Unless otherwise stated, the quantitative tests, including uniaxial compressive strength, splitting tensile strength, slump, and rebound rate, were performed three times for each mixture and testing condition. The arithmetic mean of the three measurements was used as the representative value [41,42]. During specimen preparation, cement, coarse aggregate, fine aggregate, and admixtures were first dry-mixed for 1 min. The alkali-resistant glass fibers, CNT aqueous dispersion, and the remaining mixing water were then introduced, followed by wet mixing for 5 min. The wet-mixing stage was intended to distribute the three fiber components throughout the fresh mixture and further disperse the CNT aqueous suspension within the cement paste. Subsequently, the slump test was conducted according to GB/T50080-2016, and the mixture was cast into molds with dimensions of 100 mm × 100 mm × 100 mm, followed by vibration, covering with plastic film, and storage at room temperature for 18 h. After demolding, the specimens were cured under controlled conditions at a temperature of 20 ± 2 °C and a relative humidity ≥ 95%. These standard moist-curing conditions were selected to provide a controlled and reproducible environment for comparing the 16 orthogonal mixtures and to minimize the influence of temperature and moisture fluctuations on hydration and strength development. The curing ages were 3, 7, and 28 days according to the experimental design. The specimen preparation process is illustrated in Figure 4.

**Figure 5:**
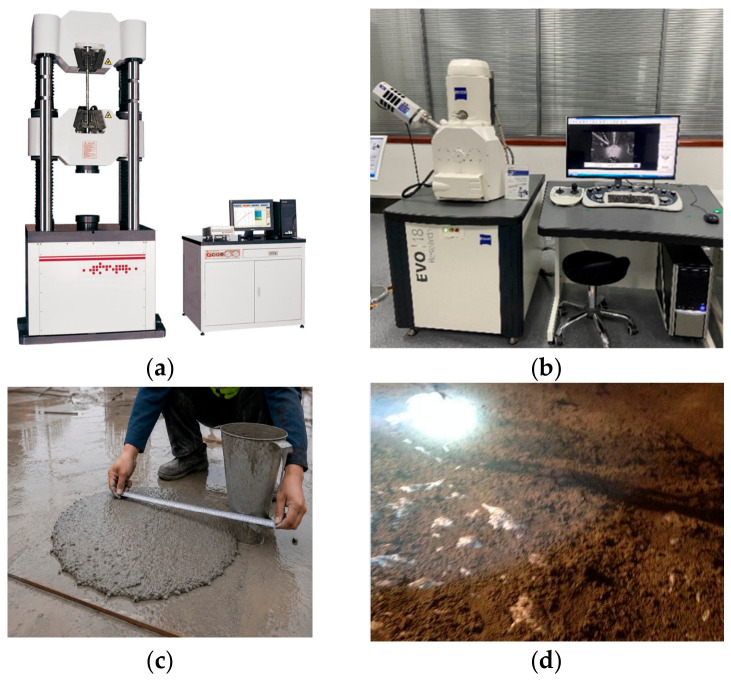
Experimental equipment used for: (**a**) universal testing machine; (**b**) SEM; (**c**) slump test; (**d**) rebound test.

After curing to the designated ages, uniaxial compressive strength, splitting tensile strength, and microscopic tests were performed [43,44,45]. The key experimental equipment used is shown in Figure 4. The rebound rate was measured in field tests: before shotcrete construction, a plastic sheet was laid under the sprayed surface to collect the rebound material, and the rebound rate was calculated as the weight of collected rebound material divided by the total weight of sprayed material. The slump was determined by filling the freshly mixed shotcrete into a truncated cone. After lifting the cone, the maximum diameter of free flow of the concrete on a glass plate was measured [46]. The uniaxial compressive strength and splitting tensile strength at 3, 7, and 28 days were measured using an electronic universal testing machine. Microscopic analysis was conducted using scanning electron microscopy (SEM). The test setups are shown in Figure 5.

## 4. Test Results and Discussions

### 4.1. Compressive Strength

**Figure 6:**
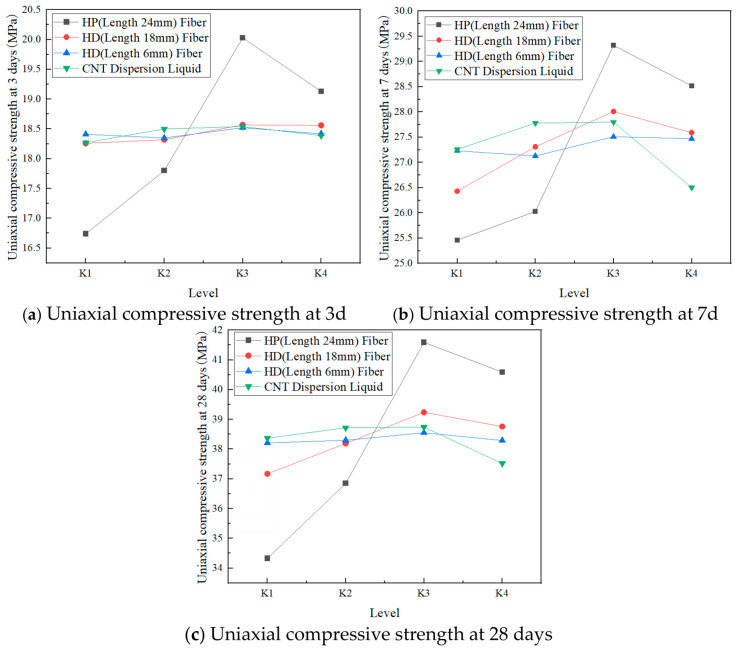
Uniaxial compressive strength of shotcrete at different level.

**Figure 7:**
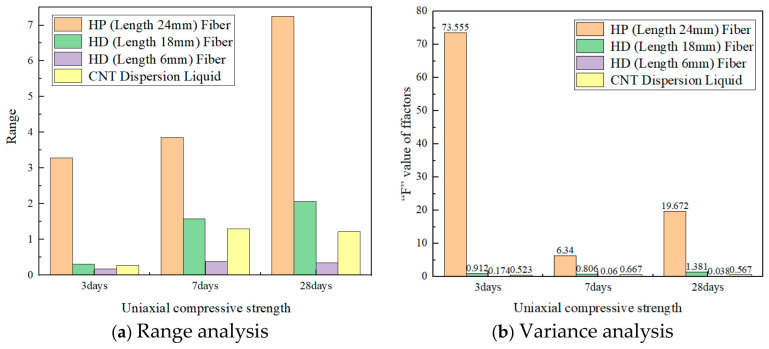
Range and variance analysis of uniaxial compressive strength.

Figure 6 presents the uniaxial compressive strength of shotcrete with different mix proportions. Figure 7 shows the range analysis and variance analysis of the compressive strength at 3, 7, and 28 days. From Figure 6 and Figure 7, it can be seen that the influence of the four factors on the uniaxial compressive strength follows the descending order. HP fiber (24 mm) content > HD fiber (18 mm) content > CNT content > HD fiber (6 mm) content.

As shown in Figure 6a and Figure 7, at 3 days, the HP fibers (24 mm) significantly enhance the compressive strength of shotcrete by forming a uniform reinforcing network. The optimal performance is achieved at an HP fiber content of 4%. The long fiber length facilitates uniform distribution in the concrete, increases tensile strength, and further improves compressive strength. Although shorter, the HD fibers (18 mm) still effectively disperse stress and enhance crack resistance. At a dosage of 2%, HD fibers provide a notable improvement in 3-day compressive strength without compromising workability. The addition of CNTs at 0.2% significantly improves the microstructure, filling microcracks and enhancing compressive strength. An appropriate CNT dosage yields the maximum strengthening effect at 3 days, while excessive addition may lead to poor dispersion and a reduced reinforcing effect.

As shown in Figure 6b and Figure 7, at 7 days, HP fibers (24 mm) continue to play a significant role, with the best compressive strength still observed at 4% content. As hydration progresses, the reinforcing effect of HP fibers becomes more pronounced, enabling better stress distribution and microstructure improvement. HD fibers (18 mm) at 2% effectively increase compressive strength while maintaining good workability. The enhancing effect of HD fibers on compressive strength is weaker than that of HP fibers, but still substantial. CNTs at 0.2% further refine the microstructure, increase compactness, and raise compressive strength. Excess CNTs may cause uneven dispersion and thus impair the strengthening effect. Previous CNT-modified cementitious composites have commonly reported beneficial effects at relatively low CNT contents, followed by a reduction in efficiency when the dosage becomes excessive because of agglomeration. The preferred CNT level of 0.20% observed in the present orthogonal analysis is consistent with this non-monotonic tendency.

As shown in Figure 6c and Figure 7, at 28 days, the reinforcing effect of HP fibers (24 mm) is most remarkable, with the optimum compressive strength again achieved at 4% content. As the hydration reaction of concrete nears completion, HP fibers are better distributed throughout the concrete, disperse stress, inhibit crack propagation, and further enhance compressive strength. HD fibers (18 mm) at 2% continue to improve compressive strength, though the increment is relatively modest. Shorter fibers provide a smaller but still significant contribution to compressive strength. CNTs at 0.2% maximize the compressive strength improvement by optimizing the microstructure. At 28 days, CNTs fully exert their effect, significantly increasing the compactness and strength of the concrete. Excessive CNT addition may lead to poor dispersion and diminish the enhancement. Therefore, the optimal mix proportion for achieving the maximum uniaxial compressive strength at 3, 7, and 28 days is α_3_β_3_γ_3_δ_3_.

### 4.2. Splitting Tensile Strength

**Figure 8:**
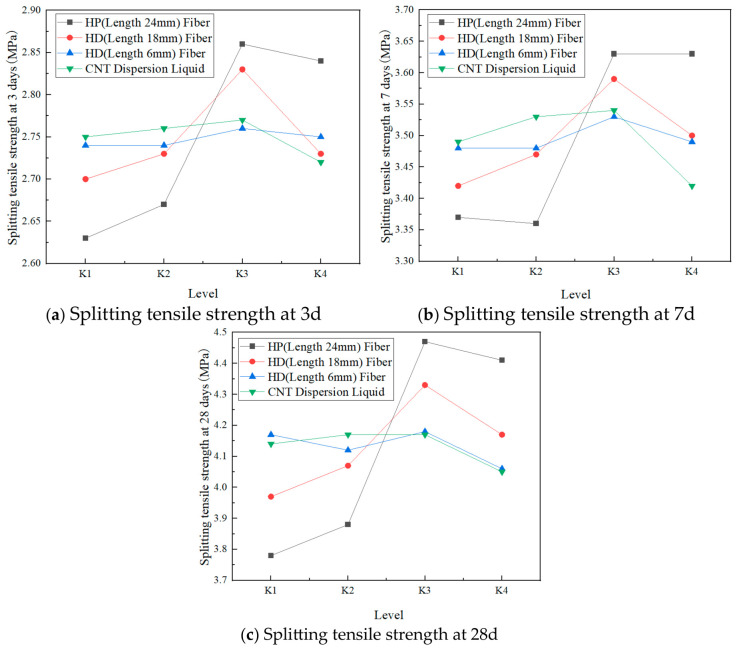
Splitting tensile strength of shotcrete at different level.

**Figure 9:**
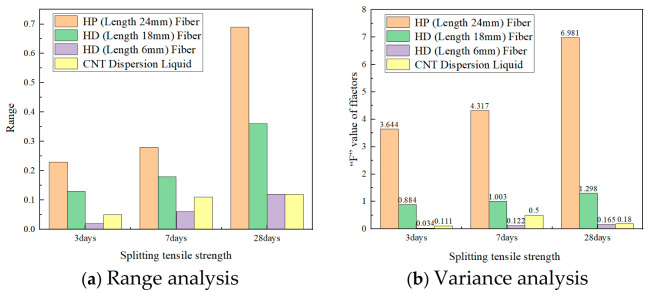
Range and variance analysis of Splitting tensile strength.

Figure 8 presents the splitting tensile strength of shotcrete with different mix proportions. Figure 9 shows the range analysis and variance analysis of the splitting tensile strength at 3, 7, and 28 days. As shown in Figure 8a and Figure 9, at 3 days, HP fibers (24 mm) effectively enhance the crack resistance and tensile strength of concrete due to their longer fiber length, with the optimal performance achieved at a dosage of 4%. At this early age, the hydration reaction of concrete is not yet complete; HP fibers primarily improve tensile strength by reducing the formation of microcracks and enhancing crack resistance. HD fibers (18 mm) at a dosage of 2% effectively improve the crack resistance and toughness of concrete without compromising its flowability and workability. CNTs at 0.2% refine the microstructure, fill microcracks, and enhance tensile strength; however, excessive CNT addition may lead to poor dispersion and diminish the strengthening effect.

As shown in Figure 8b and Figure 9, at 7 days, the reinforcing effect of HP fibers becomes more significant, with the best tensile strength again obtained at 4% content. As hydration proceeds, HP fibers better distribute stress, prevent crack propagation, and further improve tensile strength. HD fibers (18 mm) at 2% provide a favorable strengthening effect, effectively enhancing the toughness and crack resistance of concrete without affecting workability. CNTs still significantly improve the microstructure and tensile strength at 7 days; the optimal dosage remains 0.2%, while excessive addition may cause uneven dispersion and weaken the enhancement.

As shown in Figure 8c and Figure 9, at 28 days, the reinforcing effect of HP fibers reaches its maximum, particularly at a dosage of 4%, where the tensile strength is optimal. At this stage, the hydration reaction is nearly complete, and HP fibers significantly enhance tensile strength through their uniformly distributed reinforcing network. HD fibers (18 mm) at 2% continue to improve tensile strength; although their effect is slightly inferior to that of HP fibers, they still effectively increase crack resistance and toughness. CNTs also notably improve the 28-day tensile strength, with the optimal dosage of 0.2% maximizing the strengthening effect. An appropriate amount of CNTs effectively fills microcracks and increases the compactness of concrete, whereas excessive addition may impair dispersion and reduce the reinforcing efficiency. Consequently, the optimal mix proportion for achieving the maximum splitting tensile strength at 3, 7, and 28 days is α_3_β_3_γ_3_δ_3_.

### 4.3. Slump Test

**Figure 10:**
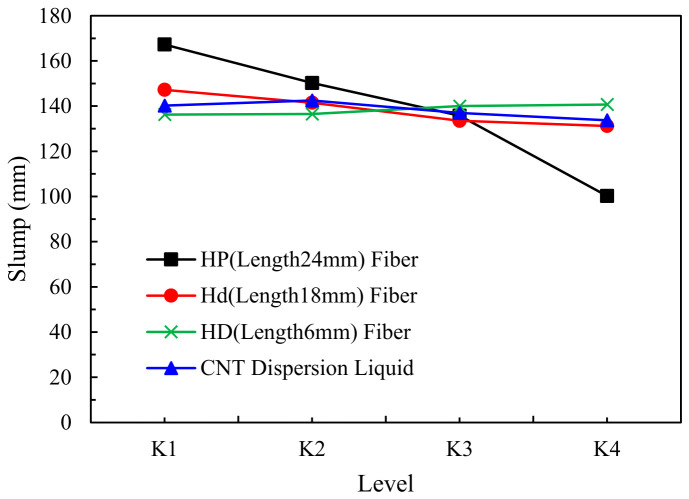
Slump of shotcrete at different level.

Figure 10 presents the slump values of shotcrete with different levels of each factor. The slump generally decreases with increasing fiber and CNT dosages, though the extent of reduction varies significantly among the four factors. Among all factors, HP fiber (24 mm) exhibits the most pronounced influence on slump. As its dosage increases from Level 1 (2%) to Level 4 (5%), the slump drops sharply from 167.25 mm to 100.25 mm, a reduction of 67 mm. This substantial decrease is attributed to the long fiber length and high aspect ratio, which enhance the physical intertwining and frictional resistance among fibers and between fibers and aggregates, thereby restricting the flowability of the fresh mixture. HD fiber (18 mm) also causes a notable decline in slump, though to a lesser extent, with values decreasing from 147.25 mm at Level 1 (1%) to 131.25 mm at Level 4 (2.5%), representing a reduction of 16 mm. This relatively moderate effect is due to the shorter fiber length, which results in less entanglement and lower resistance to flow compared to the 24 mm fibers. In contrast, HD fiber (6 mm) shows a negligible influence on slump, with values remaining nearly stable across all four levels (ranging from 136.25 mm to 140.75 mm). The minimal variation indicates that short fibers are well dispersed without significantly hindering the flowability of the fresh mixture. CNT dispersion liquid also has a limited effect on slump, with values fluctuating within a narrow range of 133.75–142.50 mm as the CNT content increases. The slight reduction at higher CNT levels may be attributed to the increased viscosity of the paste due to the high specific surface area of CNTs and the additional water introduced by the dispersion liquid. However, within the studied dosage range (0.1–0.25%), the overall impact on slump is relatively small.

The reduction in slump associated with HP-24 should not be interpreted only as a negative effect. A certain increase in cohesiveness may be beneficial for material retention on vertical or overhead surfaces. However, excessive slump loss may impair pumping, spraying continuity, fiber dispersion, and surface finishing. Therefore, the practical objective is not to maximize slump or strength independently, but to maintain sufficient consistency while achieving the required mechanical performance.

### 4.4. Rebound Rate Test

**Figure 11:**
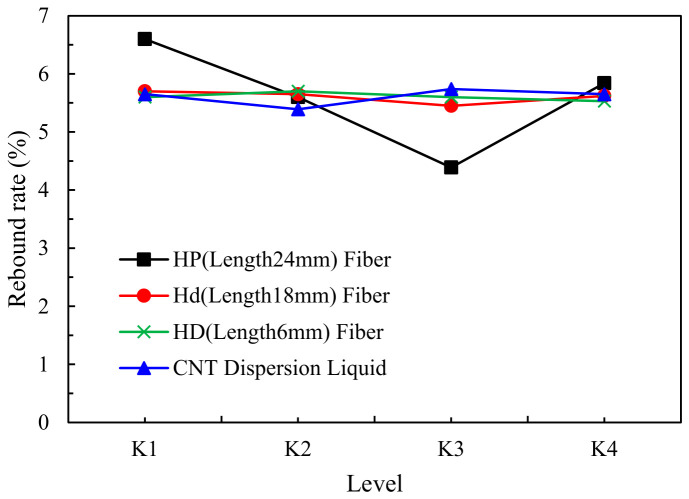
Rebound rate of shotcrete at different level.

Figure 11 presents the rebound rates of shotcrete with different levels of each factor. The rebound rate varies considerably among the four factors, with HP fiber (24 mm) showing the most distinct trend. For HP fiber (24 mm), the rebound rate decreases progressively from Level 1 (6.60%) to Level 3 (4.39%), and then increases to 5.84% at Level 4. The significant reduction from Level 1 to Level 3 indicates that the addition of long fibers effectively improves the cohesiveness of the fresh mixture, enhancing the adhesion between shotcrete and the surrounding rock surface, thereby reducing material loss during spraying. However, when the HP fiber content reaches 5% (Level 4), the rebound rate rises again. This increase is likely due to excessive fiber content causing poor dispersion and fiber agglomeration, which weakens the internal uniformity of the mixture and leads to higher rebound during the spraying process. For HD fiber (18 mm), the rebound rate shows a slight decreasing trend from Level 1 (5.70%) to Level 3 (5.45%), followed by a marginal increase to 5.62% at Level 4. However, the overall variation across all four levels is relatively small (only 0.25 percentage points), indicating that the 18 mm fiber has a limited influence on rebound rate within the studied dosage range. For HD fiber (6 mm), the rebound rate remains almost constant across all levels, fluctuating narrowly between 5.53% and 5.70%. This suggests that short fibers do not significantly affect the sprayability of shotcrete, as their small size and low entanglement tendency do not substantially alter the rheological properties of the fresh mixture. For CNT dispersion liquid, the rebound rate decreases to its minimum value of 5.39% at Level 2 (0.15% CNT), and then increases to 5.74% at Level 3 before dropping slightly to 5.65% at Level 4. The overall variation is modest (0.35 percentage points), implying that CNT content has a minor effect on rebound rate. The slight improvement at 0.15% CNT may be attributed to the enhanced cohesiveness of the paste due to the filling effect of nano-materials, while excessive CNT addition may increase the viscosity and impair the spraying performance.

The rebound rate reflects one aspect of sprayability and material utilization, but it should not be interpreted independently of slump and mixture cohesiveness. A mixture with insufficient cohesion may exhibit high rebound because coarse particles are not retained on the receiving surface, whereas a mixture with excessively low workability may also show unstable discharge and fiber accumulation. The lowest rebound value therefore does not by itself establish the most suitable field mixture unless pumping, nozzle behavior, adhesion, and layer build-up are also satisfactory.

### 4.5. Microstructural Analysis

**Figure 12:**
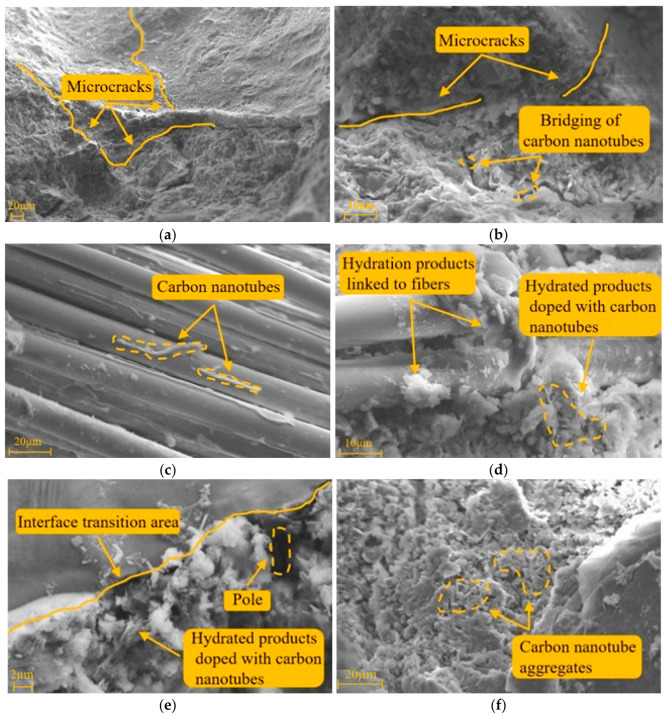
SEM images of CNT-reinforced hybrid AR-GFRC: (**a**) internal microcracks; (**b**) bridging of carbon nanotubes; (**c**) hydration products on fiber surface; (**d**) hydration between fibers and surrounding concrete; (**e**) interfacial transition zone; (**f**) agglomerates of carbon nanotubes.

To elucidate the underlying reinforcement mechanisms, SEM was performed on the shotcrete specimens. The SEM images are presented in Figure 12, revealing the microstructural features including internal microcracks, CNT bridging, fiber surface hydration products, interfacial transition zone characteristics, and CNT dispersion states.

As shown in Figure 12a, microcracks are observed within the cementitious matrix. These microcracks are primarily generated during the hardening process due to autogenous shrinkage or external loading. However, the presence of fibers and CNTs across these microcracks effectively restrains their further propagation. As shown in Figure 12a, several local microcracks are visible within the examined cementitious matrix. Some cracks appear relatively narrow and discontinuous within this particular field of view. These local observations are consistent with a possible crack-restraining role of the reinforcing components; however, the image does not provide a quantitative measurement of microcrack density, crack-width distribution, or overall cracking throughout the specimen. Figure 12b clearly demonstrates the bridging effect of carbon nanotubes across microcracks. CNTs are observed to span the crack openings, connecting the two crack faces. This nano-scale bridging action transfers stress across the cracked region and prevents the widening of microcracks. The strong interfacial bonding between CNTs and the surrounding hydration products ensures that the bridging effect remains effective under tensile loading, thereby contributing to the enhanced tensile strength observed at the macroscopic level.

Figure 12c shows the surface morphology of a glass fiber after hydration. A dense layer of hydration products, including C-S-H gel and ettringite-like phases, is uniformly deposited on the fiber surface. This indicates a favorable chemical affinity between the fiber surface and the cementitious matrix, which promotes mechanical interlocking and chemical bonding. The well-hydrated fiber surface also suggests that the fibers are effectively wetted by the cement paste during mixing, ensuring good bond quality. Figure 12d presents the interfacial region between a fiber and the surrounding hydrated concrete matrix. The fiber is closely encapsulated by hydration products, with no visible gaps or debonding along the interface. The dense and continuous contact at the fiber–matrix interface indicates that the fiber is well integrated into the cementitious system. This strong interfacial bond is essential for stress transfer from the matrix to the fibers, which directly contributes to the improved mechanical performance of the composite.

Figure 12e depicts the interfacial transition zone (ITZ) between coarse aggregate and the cement paste. The ITZ appears relatively dense and compact, with hydration products filling the pores around the aggregate surface. The improved compactness of the ITZ is attributed to the combined effects of CNTs and short fibers, which refine the pore structure and reduce the wall effect commonly observed in conventional concrete. A denser ITZ reduces the permeability and enhances the overall durability of shotcrete. Figure 12f reveals the presence of CNT agglomerates in certain local areas. Despite the use of aqueous dispersion, a small number of CNTs remain entangled and form clusters. These agglomerates act as weak spots within the matrix, potentially causing stress concentration and reducing the expected reinforcing efficiency. This observation explains the diminishing enhancement effect when CNT content exceeds the optimal dosage (0.2%), as excessive CNTs tend to agglomerate due to van der Waals forces, leading to non-uniform dispersion and impaired microstructure. The proposed formulation contains CNTs and three types of alkali-resistant glass fibers, and therefore its initial material cost is expected to be higher than that of conventional shotcrete. In addition to the direct cost of the reinforcing components, the use of a multi-component system may increase requirements for material storage, dosage control, mixing consistency, and quality assurance. CNT dispersion stability may also require stricter control during production, which could further increase implementation complexity.

## 5. Conclusions

This study developed a cross-scale structured hybrid fiber-reinforced shotcrete by incorporating alkali-resistant glass fibers such as HP 24 mm, HD 18 mm, HD 6 mm and carbon nanotubes into a conventional shotcrete matrix. Based on orthogonal experimental results, the following conclusions are drawn:

(1) The optimal mix proportion is HP fiber (24 mm) at 4%, HD fiber (18 mm) at 2%, HD fiber (6 mm) at 2%, and CNT at 0.2%. This combination, identified through range and variance analyses, does not exactly match any individual test run, demonstrating the effectiveness of orthogonal design.

(2) Among the four factors, HP fiber (24 mm) exerts the dominant influence on both compressive and tensile strengths, followed by HD fiber (18 mm), CNT, and HD fiber (6 mm). The optimal mix achieves a 28-day compressive strength of 43.53 MPa and splitting tensile strength of 4.85 MPa.

(3) Slump is primarily governed by HP fiber content, which reduces flowability from 167.25 mm to 100.25 mm as dosage increases from 2% to 5%. The rebound rate reaches its minimum of 4.39% at 4% HP fiber, with variance analysis confirming that HP fiber is the only statistically significant factor affecting rebound rate.

(4) SEM observations reveal a multi-scale reinforcing mechanism: long fibers suppress macroscopic cracks, CNTs bridge microcracks at the nano-scale, hydration products densely cover fiber surfaces and the interfacial transition zone, and CNT agglomerates appear at excessive dosages, explaining the diminishing returns beyond 0.2% CNT.

(5) Within the investigated factor ranges and controlled experimental conditions, the selected formulation exhibited a favorable balance among mechanical strength, workability, and rebound behavior. This formulation should be regarded as a laboratory-scale candidate for further evaluation rather than as a field-validated solution for underground support. Its suitability for high-geostress, water-rich, or high-temperature tunnel environments must be verified through pilot-scale spraying, in situ structural testing, durability assessment, and long-term field monitoring.

(6) Unreinforced, fiber-only, CNT-only, and complete hybrid control mixtures should be included to distinguish the individual and combined contributions of the reinforcing components. Interaction-oriented factorial or response-surface designs are also required to quantitatively evaluate fiber–fiber and fiber–CNT interactions.

## Figures and Tables

**Table 1:** Physical and mechanical properties of cement.

Density (g/cm^3^)	Fineness (%)	Specific Surface Area (m^2^/kg)	Standard Consistency (g)	Soundness (mm)	Setting Time (min)		Flexural Strength (MPa)		Compressive Strength (MPa)	
					Initial	Final	3d	28d	3d	28d
3.15	0.6	349	25	Qualified	151	210	5.7	8.8	28.5	51.1

**Table 2:** Technical parameters of alkali-resistant glass fibers.

Fiber Type	Monofilament Diameter (μm)	Number of Monofilaments	Specific Gravity (g/cm^3^)	Softening Point (°C)	Elastic Modulus (GPa)	Tensile Strength (MPa)	ZrO_2_ (%)
HD	14	2400	2.68	860	72	1700	≥16.5
HP	19	600	2.68	860	72	1700	≥16.5

**Table 3:** Mix proportion of shotcrete (kg/m^3^).

Cement	Water	Coarse Aggregate	Fine Aggregate	Accelerator	Superplasticizer	Silica Fume
480	233	850	785	24	1.44	33.6

**Table 4:** Orthogonal design array.

No.	Factor α	Factor β	Factor γ	Factor δ
1	1	1	1	1
2	1	2	2	2
3	1	3	3	3
4	1	4	4	4
5	2	1	2	3
6	2	2	1	4
7	2	3	4	1
8	2	4	3	2
9	3	1	3	4
10	3	2	4	3
11	3	3	1	2
12	3	4	2	1
13	4	1	4	2
14	4	2	3	1
15	4	3	2	4
16	4	4	1	3

## Data Availability

The original contributions presented in the study are included in the article, further inquiries can be directed to the corresponding authors.
